# Revealing the Effects of Zinc Sulphate Treatment on Melatonin Synthesis and Regulatory Gene Expression in Germinating Hull-Less Barley through Transcriptomic Analysis

**DOI:** 10.3390/genes15081077

**Published:** 2024-08-15

**Authors:** Yufeng Guo, Guoqiang Zhang, Zhenghong Li, Xueyi Liao, Wu Sun, Xinhao Jiang

**Affiliations:** School of Biological and Food Engineering, Anhui Polytechnic University, Wuhu 241000, China; gyf15765975285@163.com (Y.G.); 15310458040@163.com (Z.L.); lxy564118298@gmail.com (X.L.); 15855663002@163.com (W.S.); 15353333364@163.com (X.J.)

**Keywords:** abiotic stress, zinc sulphate, hull-less barley, melatonin, transcriptome

## Abstract

This study investigated the transcriptomic mechanisms underlying melatonin accumulation and the enhancement of salt tolerance in hull-less barley seeds subjected to zinc sulphate stress. Following zinc sulphate treatment, hull-less barley seeds demonstrated increased melatonin accumulation and improved salt tolerance. Through transcriptome analysis, the study compared gene expression alterations in seeds (using the first letter of seed, this group is marked as ‘S’), seeds treated with pure water (as the control group, is marked as ‘C’), and germinated seeds exposed to varying concentrations of zinc sulphate (0.2 mM and 0.8 mM, the first letter of zinc sulphate, ‘Z’, is used to mark groups ‘Z1’ and ‘Z2’). The analysis revealed that 8176, 759, and 622 differentially expressed genes (DEGs) were identified in the three comparison groups S.vs.C, C.vs.Z1, and C.vs.Z2, respectively. Most of the DEGs were closely associated with biological processes, including oxidative-stress response, secondary metabolite biosynthesis, and plant hormone signaling. Notably, zinc sulphate stress influenced the expression levels of Tryptophan decarboxylase 1 (*TDC1*), Acetylserotonin O-methyltransferase 1 (*ASMT1*), and Serotonin N-acetyltransferase 2 (*SNAT2*), which are key genes involved in melatonin synthesis. Furthermore, the expression changes of genes such as Probable WRKY transcription factor 75 (*WRKY75*) and Ethylene-responsive transcription factor ERF13 (*EFR13*) exhibited a strong correlation with fluctuations in melatonin content. These findings contribute to our understanding of the mechanisms underlying melatonin enrichment in response to zinc sulphate stress.

## 1. Introduction

The soil ecosystem is an open and complex system, and its pollution directly affects environmental quality, food security, and public health [1]. In recent years, with the acceleration of urbanisation and industrialisation, soil pollution has shown diverse, complex, and regional development [2]. The sources and effects of zinc, as a key chemical stressor, are particularly significant. Natural soil-forming matrices are the main source of Zn, but anthropogenic activities, such as smelting and mining, also contribute significant amounts of Zn to the soil. In addition, the use of Zn-containing pesticides/fertilisers and livestock manure inputs also increases soil Zn levels [3]. These anthropogenic sources of Zn are highly mobile and can easily over-accumulate in plants, thereby causing toxic effects and metabolic disorders [4]. Although Zn deficiencies are more common, the toxicity of excess Zn to plants should not be overlooked, especially in acidic and sludge-amended soils. Zinc sulphate (ZnSO_4_), a commonly used zinc fertiliser, can easily lead to excessive soil zinc levels when used improperly, which can have a toxic effect on plants.

Barley is a historic highland cereal that ranks fourth in the world in terms of production and acreage [5] and is mostly used for feed and beer making, among other things, with a small portion being used for direct human consumption [6]. A special variety of barley is widely planted in the Tibetan region of China and has attracted much attention in recent years because it is cold- and drought-resistant and rich in nutrients. It is especially favoured by modern people due to its richness in protein, dietary fibre, and β-glucan, which are extremely beneficial to human health [7].

In the plant kingdom, melatonin is a widely distributed indole-like substance that is found in several parts of the plant, especially the seeds, roots, and leaves [8,9]. It is noteworthy that barley, as an important crop, is particularly rich in melatonin in its seeds, at levels even higher than 5 ng·g^−1^, and this content further increases under specific physiological conditions, such as seed germination and salt-stress environments [10]. Melatonin is multifunctional and has similar effects to growth hormones, stimulating seed germination, plant growth, and adventitious root formation [11]. In addition, melatonin is a crucial plant metabolite and a major regulator of abiotic stress [12,13,14]. For example, under salt-stress conditions, pretreatment with exogenous melatonin can significantly enhance the enzyme activity of plants and activate more differentially expressed genes, thereby enhancing the salt-tolerance mechanism of crops such as okra [15]. Similarly, under Cd stress, melatonin can be regulated by heat shock factors (e.g., *HsfA1a*) to increase synthesis, thereby enhancing resistance to Cd stress in plants such as tomato [16].

In recent years, zinc sulphate has shown its unique value as a multifunctional compound in multiple fields. In the food industry, food-grade zinc sulphate has become an indispensable component in dairy products and infant formula, providing the necessary zinc element for the human body and promoting healthy growth and development [17,18]. In the field of agriculture, zinc sulphate also plays an important role. It can promote plant photosynthesis, enhance plant growth vitality, and improve yield and quality [19]. Zinc sulphate also plays an important role in plant physiological processes, especially in the synthesis of melatonin. Research has shown that the application of zinc sulphate can increase the content of melatonin in plants. For example, when ZnSO_4_ was applied to lupin, it was able to increase the endogenous melatonin content [20]. In barley, the application of 1 mM zinc sulphate to the roots (in vivo or in slices) significantly increased melatonin content after 7 days of germination [21]. Similarly, a study by Xu et al. [22] revealed that in the case of hull-less barley subjected to ZnSO_4_ stress, an enhancement in endogenous melatonin levels was observed, although a certain degree of toxicity was also produced.

Although there have been initial reports on the effect of ZnSO_4_ on plant melatonin content, there is still a lack of in-depth studies on its specific mechanism of action in barley at the transcriptomic level. Transcriptomics, as an important tool in the study of the regulation of gene expression in plants under abiotic stress [23,24,25,26], is able to reveal the changes in the expression of genes related to melatonin synthesis in barley under ZnSO_4_ treatment and to elucidate its regulatory mechanism. Therefore, this study aims to use transcriptomic analysis methods to systematically investigate the effects of ZnSO_4_ on barley melatonin synthesis and related gene expression, with the aim of revealing the intrinsic relationship between ZnSO_4_ and melatonin.

## 2. Materials and Methods

### 2.1. ZnSO_4_ Stress Germination in Barley

**Figure 1:**
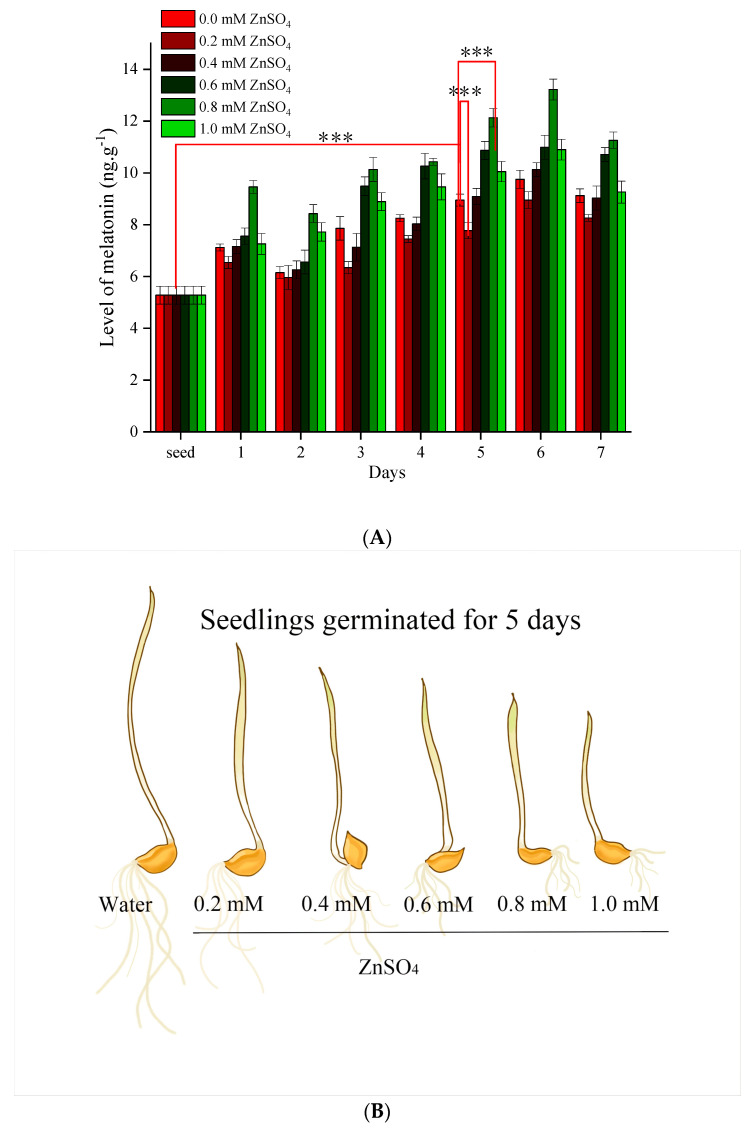
(**A**) Changes in melatonin content of barley under ZnSO_4_ stress treatments at different concentrations of ZnSO_4_. (**B**) Growth of barley seedlings 5 days after germination under different concentrations of ZnSO_4_ stress (gradually increasing ZnSO_4_ concentration from left to right) (data from Xu et al. [19]. *** The difference in means has a significance level of 0.01).

‘Zangqing 2000’ hull-less barley seed (*Hordeum vulgare* L.), which has good salt and alkali tolerance, was provided by the Academy of Agricultural and Animal Husbandry Sciences of the Tibet Autonomous Region. The seed germination treatment was carried out according to Xu et al.’s method [22]. The seeds were sterilised with 1% sodium hypochlorite solution for 10 min, rinsed with deionised water until the seeds were odourless, and then soaked in deionised water for 10 h to hydrate the seeds. Seeds were subsequently dried using absorbent paper and then placed in germination cassettes containing varying concentrations of zinc sulphate solution (0, 0.2, 0.4, 0.6, 0.8, and 1.0 mM). The germination process was conducted over a period of 7 days (25 °C, protected from light) to assess the melatonin content. Based on the melatonin content (Figure 1A), the most suitable four groups of samples were selected: (A) seeds soaked in pure water for 10 h (set as ‘S’); (B) seeds germinated after treatment with pure water (set as ‘C’); (C) seeds germinated after treatment with 0.2 mM ZnSO_4_ (set as ‘Z1’); and (D) seeds germinated after treatment with 0.8 mM ZnSO_4_ (set as ‘Z2’). The collected complete seedlings were immediately frozen in liquid nitrogen and stored at −80 °C for transcriptomic analysis. Three biological replicates were performed for each experiment.

### 2.2. Extraction and Determination of Melatonin

Melatonin content in barley was determined according to Zhang et al.’s method [10]. Initially, 2 g of the sample was ground in liquid nitrogen and then dissolved in 5 mL of chloroform for overnight extraction at 4 °C. Subsequently, the sample was centrifuged at 4 °C (10,000× *g*, 15 min), and the chloroform was evaporated with nitrogen until dry. The extraction solution was prepared using 1 mL of 42% methanol solution and filtered through a 0.45 µm filter membrane. Following this, 400 μL of all prepared samples were subjected to melatonin analysis using high-performance liquid chromatography (SPD-20A Prominence, Shimadzu Co., Ltd., Kyoto, Japan) with a Shim pack VP-ODS chromatography column (3 μm, 4.6 × 150 mm, Shimadzu). Gradient elution was carried out from 42% methanol to 50% methanol with 0.1% formic acid over 27 min, followed by an isobaric elution with 50% methanol and 0.1% formic acid for 18 min at a flow rate of 0.15 mL·min^−1^. Detection of melatonin was performed at 280 nm excitation and 348 nm emission.

### 2.3. Transcriptome Sequencing and De Novo Assembly

Total RNA was extracted from the liquid nitrogen–frozen samples, and the qualified RNA samples were sent to Shanghai WikiBiotech for library construction. These libraries were subjected to paired-end (PE) sequencing using next-generation sequencing (NGS) technology and the advanced Illumina sequencing platform. At the same time, a strict data analysis process was used to assess and screen the quality of the sequencing data, removing low-quality sequences and noisy data in order to obtain high-quality sequencing results. Sringtie [27] was used for de novo assembly of the transcripts.

### 2.4. Differentially Expressed Gene (DEG) Analysis and Functional Enrichment Analysis

The transcripts per kilobase per million mapped reads (FPKM) values were calculated using RESM v1.3.3 software [28]. Differential analysis of gene expression was performed using DESeq, and the conditions for screening the differentially expressed genes were as follows: |log2FoldChange| > 1 and a significance of *p* < 0.05. The differentially expressed genes (DEGs) were analysed using Gene Ontology (GO) and the Kyoto Encyclopedia of Genes and Genomes (KEGG).

### 2.5. Statistical Analyses

The data were statistically analysed using SPSS software (version 26.0) and are presented as mean ± standard deviation; the differential genes were screened via the expression of the multiplicity of variance |log2FoldChange| > 1 and the significance *p* < 0.05. Each treatment was repeated three times.

## 3. Results

### 3.1. Selection of Barley Samples Stressed with Different Concentrations of ZnSO_4_

In our previous study (published, see Xu et al. [22]), we observed that under different concentrations of ZnSO_4_ stress treatments, the melatonin content in barley seedlings showed a gradual increase, and the seedlings became progressively shorter as the sprouting time increased (as shown in Figure 1B), suggesting that the concentration of ZnSO_4_ selected for the experiments (0.2~1.0 mM) exerted a toxic effect on barley. However, the laboratory environment was not absolutely sterile, and the seeds began to be infested with moulds by day six of germination. Thus, we selected seedlings that would germinate on day five for the subsequent transcriptome analysis. Interestingly, on the fifth day of barley sprout germination, we found that the melatonin content in the barley seedlings under the 0.2 mM ZnSO_4_ treatment reached the lowest level of (7.77 ± 0.31) ng·g^−1^, whereas the melatonin content reached the highest level of (12.13 ± 0.36) ng·g^−1^ under the 0.8 mM ZnSO_4_ treatment (Figure 1A). In order to further investigate the changes in the melatonin-related gene expression in barley under different concentrations of ZnSO_4_ stress, we carefully selected the following samples for the subsequent transcriptome analysis: seeds (using the first letter of seed, this group is marked as “S”) and Tibetan green 2000 seedlings (pure water, as the control group, is marked as “C”, and the first letter of zinc sulphate, “Z”, is used to mark groups “Z1” and “Z2”). These samples were treated with pure water and ZnSO_4_ at concentrations of 0.2 and 0.8 mM and germinated for 5 days. In the subsequent analyses, we focused on the following three sets of comparisons: the comparison of S with C; the comparison of C with Z1; and the comparison of C with Z2.

### 3.2. Transcriptome Analysis and Differential Gene (DEG) Identification

**Figure 2:**
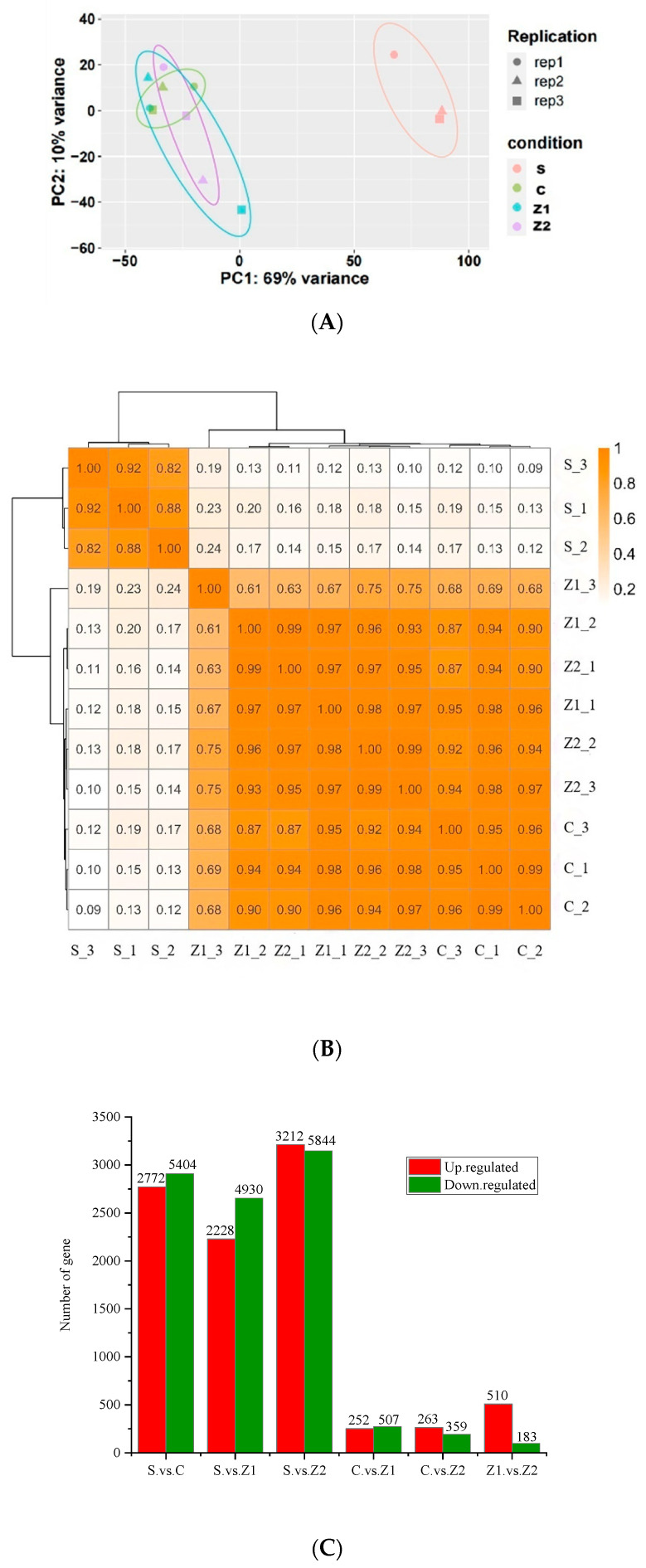
Differential gene (DEG) identification. (**A**) PCA analysis plot of differentially expressed genes detected in comparison groups S_C_Z1_Z2; (**B**) the number of up- and down-regulated genes in each comparison group (red indicates the number of up-regulated genes and blue-green indicates the number of down-regulated genes); (**C**) correlation analysis between S_C_Z1_Z2.

To further understand the molecular response mechanisms during melatonin biosynthesis in barley under zinc sulphate stress, we performed transcriptome sequencing analysis of components from four different treatments. A total of 58,815 expressed genes were identified (Table S1), of which 11,132 exhibited significant differential expression (DEGs) (Table S2). To further visualise the differences between the four fractions, we employed principal component analysis (PCA). As shown in Figure 2A, PC1 and PC2 contributed 69% and 10% of the total variance, respectively, which fully demonstrated the major differences among the components. It can be clearly seen in the figure that the three biological replicates of the four components are closely clustered, which proves the reliability and consistency of the obtained transcriptome data. It is worth mentioning that there was a significant separation between S and the other three groups, which implied that the seeds were significantly different from the germinated seeds treated with ZnSO_4_ in terms of gene expression levels. In contrast, the three groups C, Z1, and Z2 exhibited relatively small differences in the PCA plots due to the fact that they had similar germination patterns and were treated with different concentrations of ZnSO_4_, and a certain degree of differentiation could still be observed. To further validate these observations, we also performed correlation analyses. As shown in Figure 2B, there were significant differences in gene expression patterns among the four groups of samples, which further confirmed that our transcriptome data were reliable and reflective of the molecular response of melatonin in barley under ZnSO_4_ stress.

We identified the differentially expressed genes (DEGs) in each comparison group based on the criteria of expression difference multiplicity|log2FoldChange| > 1 and the significance *p* < 0.05. The number of DEGs identified ranged from 622 to 9056 in the six comparison groups (S.vs.C, S.vs.Z1, S.vs.Z2, C.vs.Z1, C.vs.Z2, and Z1.vs.Z2). Notably, the highest number of DEGs identified in the S.vs.Z2 comparison group was 9056, including 3212 up-regulated and 5844 down-regulated genes. Conversely, the smallest differences in gene expression were found in C.vs.Z2, with only 263 up-regulated genes and 359 down-regulated genes (Figure 2C). Further analysis revealed that the number of DEGs was significantly higher in the S.vs.C, S.vs.Z1, and S.vs.Z2 comparison groups than in the C.vs.Z1, C.vs.Z2, and Z1.vs.Z2 comparison groups (See Figure S1 for the relevant Upset diagram). Meanwhile, the number of down-regulated genes in these comparative groups was significantly higher than that of the up-regulated genes. In order to investigate the changes in melatonin-related genes and pathways in barley under ZnSO_4_ stress, we mainly conducted transcriptomic analysis on three comparative groups: S.vs.C, C.vs.Z1, and C.vs.Z2.

### 3.3. GO and KEGG Pathway Enrichment Analysis

**Figure 3:**
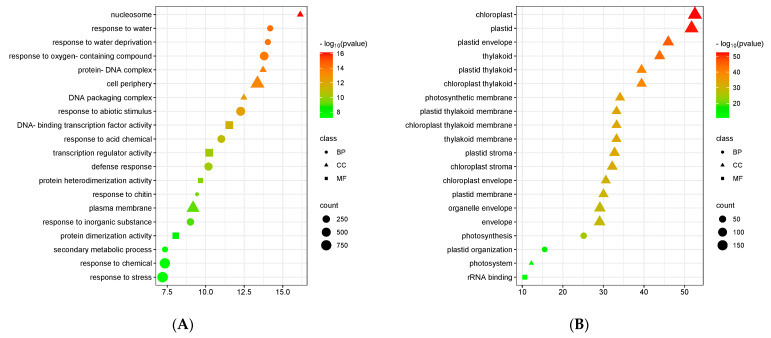

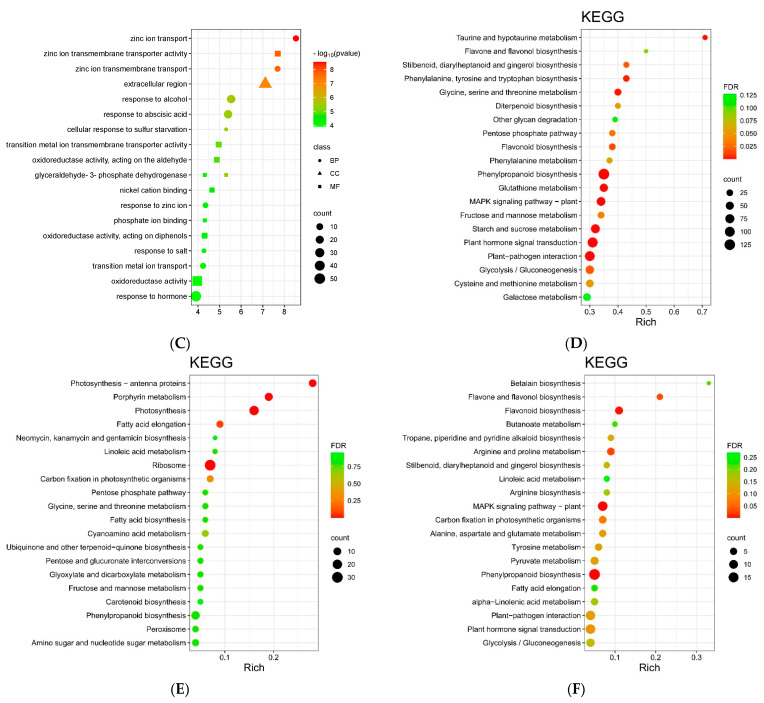
GO and KEGG enrichment analysis plots. (**A**) Bubble plot of GO enrichment analysis of differentially expressed genes in S.vs.C; (**B**) bubble plot of GO enrichment analysis of differentially expressed genes in C.vs.Z1; (**C**) bubble plot of GO enrichment analysis of differentially expressed genes in C.vs.Z2; (**D**) KEGG enrichment analysis of differentially expressed genes in S.vs.C; (**E**) KEGG enrichment of differentially expressed genes in C.vs.Z1 analysis; (**F**) KEGG enrichment analysis of differentially expressed genes in C.vs.Z2. The top 20 GO term entries and the top 20 KEGG pathways with the smallest FDR value, i.e., the most significant enrichment, were selected for display, and the closer to zero the FER or FDR, the more significant the enrichment.

In the S.vs.C comparison group, we detected the highest number of differentially expressed genes (DEGs), totalling 8176, with 2772 up-regulated genes and up to 5404 down-regulated genes (Figure 2C). Relatively, 759 and 622 DEGs were detected in the two comparison groups of C.vs.Z1 and C.vs.Z2, respectively; 252 up-regulated genes and 507 down-regulated genes were found in C.vs.Z1; and 263 up-regulated genes and 359 down-regulated genes were found in C.vs.Z2. Through GO analysis, we found that the DEGs in the S.vs.C comparison group were particularly enriched in biological processes such as the stress response, chemical response, plasma membrane, cell periphery, response to oxygenated compounds, and response to abiotic stimuli (Figure 3A and Table S3). In contrast, in the C.vs.Z1 comparison group, we found a significant enrichment of biological processes such as chloroplasts, plastids, plastid envelopes, cysts, photosynthesis membranes, and organelle envelopes (Figure 3B and Table S4). Biological processes such as response to hormones, oxidoreductase activity, response to abscisic acid, alcohol response, extracellular zone activity, zinc ion transmembrane transporter activity, and zinc ion transport were significantly enriched in the C.vs.Z2 comparison group (Figure 3C and Table S5).

In order to further investigate the important biological pathways of melatonin during barley germination and under different concentrations of ZnSO_4_ stress, we analysed the KEGG enrichment of DEGs in the S.vs.C, C.vs.Z1, and C.vs.Z2 groups. The biological pathways of phenylpropanoid biosynthesis, phytohormone signalling, plant MAPK signalling, glutathione metabolism, starch and sucrose metabolism, plant–pathogen interactions, and glycolysis/glycolysis were the top pathways that were more enriched in the S.vs.C group (Figure 3D). In contrast, processes such as ribosomes, porphyrin metabolism, antenna proteins of photosynthesis, and photosynthesis were significantly enriched in the C.vs.Z1 group (Figure 3E). Biological pathways such as phenylpropanoid biosynthesis, plant MAPK signalling, flavonoid biosynthesis, amino acid metabolism (including arginine, proline, and tyrosine), phytohormone signalling, and plant–pathogen interactions were the main ones enriched in the C.vs.Z2 comparison (Figure 3F).

### 3.4. Screening of Genes Related to Melatonin Synthesis

**Figure 4:**
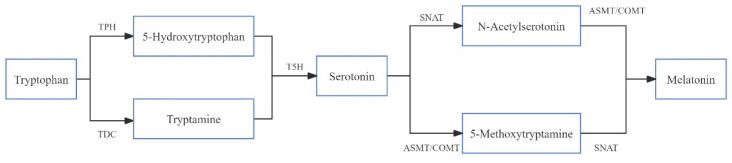
Expression of enzymes involved in the melatonin synthesis pathway in plants. TDC: L-tryptophan decarboxylase; TPH: tryptophan hydroxylase; T5H: tryptamine 5-hydroxylase; SNAT: serotonin N-acetyltransferase; ASMT: acetylserotonin O-methyltransferase; COMT: caffeic acid 3-O-methyltransferase.

Melatonin synthesis begins with tryptophan, which is converted by four enzymatic reactions [29]. These four enzymatic reactions involve six key enzymes [30]: tryptophan decarboxylase (TDC), tryptamine 5-hydroxylase (T5H), tryptophan hydroxylase (TPH), N-acetyltransferase (SNAT), N-acetylserotonin methyltransferase (ASMT), and caffeic acid O-methyltransferase (COMT) (Figure 4). We attempted to identify all genes annotated as encoding these six enzymes in order to further identify the melatonin synthesis pathway in barley under ZnSO_4_ stress at the transcriptional level. It is interesting that the encoding enzymes TDC (1 gene), ASMT (1 gene), and SNAT2 (1 gene) have different expressions in S, C, and Z1 (Table 1). However, we were unable to find any genes encoding T5H, COMT, and TPH, and there was no expression of related genes in Z2 (Table 1).

### 3.5. Transcription Factors

**Figure 5:**
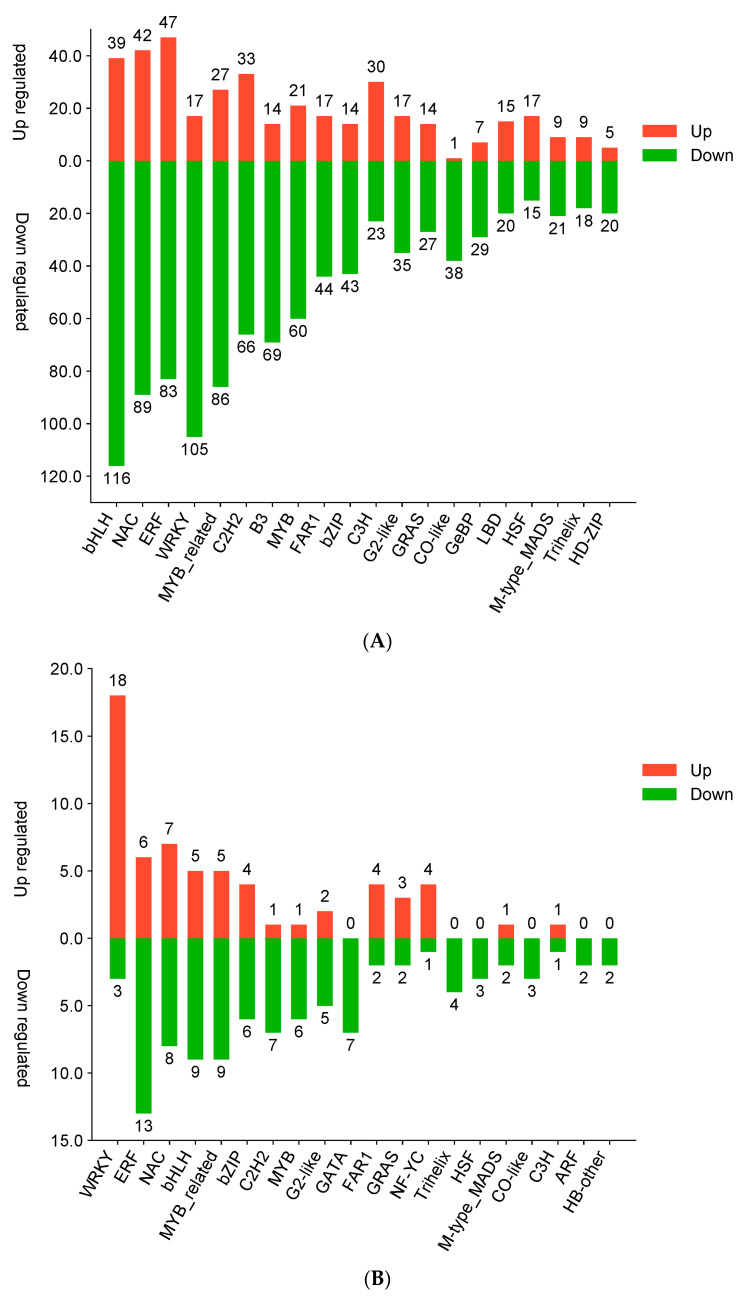

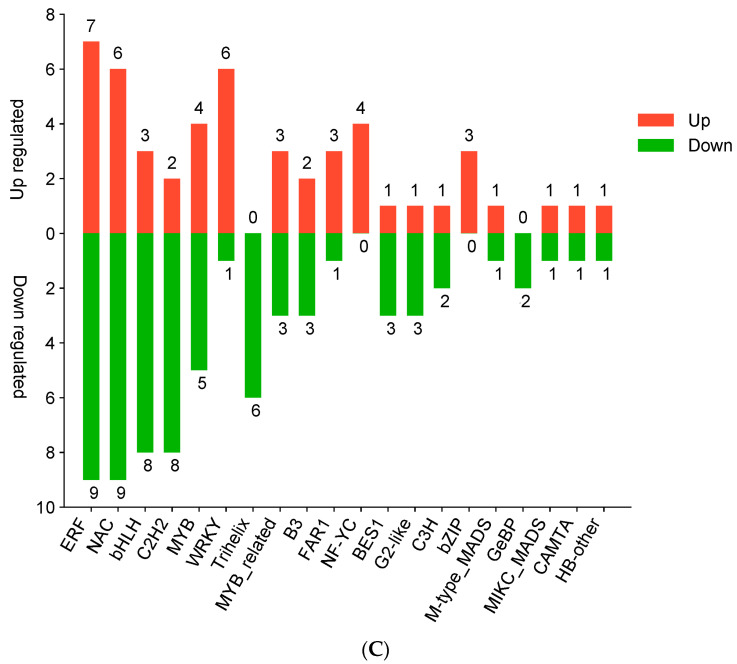
Differential transcription factor bar graphs. (**A**) Distribution of the number of up- and down-regulated TFs in S.vs.C; (**B**) distribution of the number of up- and down-regulated TFs in C.vs.Z1; (**C**) distribution of the number of up- and down-regulated TFs in C.vs.Z2. Horizontal coordinates are different transcription factor families, and vertical coordinates are the number of genes falling into that transcription factor family.

We performed a detailed analysis of the detected transcription factors (TFs). The results of the analysis revealed that a total of 1768 TFs showed differential expression in the S.vs.C group, of which 512 TFs were up-regulated, while 1256 TFs were down-regulated (Figure 5A). Further analysing the C.vs.Z1 and C.vs.Z2 groups, we found that 176 and 133 TFs showed differential expression, respectively. In the two groups, the number of up-regulated TFs was 69 and 55, respectively, while the number of down-regulated TFs was 107 and 78, respectively (Figure 5B,C). Notably, some specific TF families, such as bHLH, ERF, NAC, WRKY, MYB-related, and MYB, showed particular sensitivity under zinc sulphate stress. The genes of these TF families were generally significantly down-regulated in the S.vs.C comparison. In contrast, between the Z1 and Z2 ZnSO_4_ concentrations, the number of TFs was higher in the Z1 group than in the Z2 group, and the number of down-regulated TFs was significantly higher than that of the up-regulated TFs in both the Z1 and Z2 groups.

In addition to this, we found that one MYB-related, one MYB, six WRKY, four ERF, and four NAC genes were significantly up-regulated in the comparison of C.vs.Z1 (Table S6). We also observed the significant down-regulation of two NAC, two BHLH, three MYB-related, and two bZIP genes. Six ERF, one NAC, one BHLH, one MYB-related, and two MYB genes were significantly up-regulated in the C.vs.Z2 comparison. However, one ERF, two NAC, two BHLH, two MYB-related, and two MYB genes were also significantly down-regulated (Table S6).

## 4. Discussion

Environmental stresses, especially salt stress, have a significant effect on seed germination. Seed germination was reduced under salt-stress conditions, and root and shoot lengths were short relative to the control. This is due to the adverse effects, such as osmotic stress, oxidative stress, and ionic toxicity, triggered by salt stress [31], all of which can create obstacles to the normal growth of crops. For example, common crops, such as wheat and maize, have stunted growth under salt stress. However, some saline plants are able to grow well in such environments, thanks to their unique salt-tolerance mechanisms [32]. As a highly salt-tolerant crop, barley has become the focus of many researchers in recent years. With the help of advanced proteomics and transcriptomics technologies, these researchers have deeply explored the salt-tolerance mechanism of barley with a view to revealing its intrinsic salt-tolerance genes and molecular mechanisms [10,33].

Salt stress affects plants in many ways, and it triggers a series of complex biological responses. These responses include phenylpropanoid biosynthesis, phytohormone signalling, regulation of transcription factors, stress responses, and key processes such as amino acid biosynthesis and carbohydrate metabolism [34,35,36]. These processes are critical for plant response to salt stress and involve the expression of multiple salt-tolerant genotypes [37]. Given the central role of these salt-tolerance genes in plant response to salt stress, we hypothesised that there was a close and indispensable link between them and melatonin synthesis. This provided us with important clues that allowed us to delve into the role of melatonin in the mechanism of salt tolerance in plants. Based on transcriptomic analysis, we compared the gene expression differences between different comparison groups (S.vs.C, C.vs.Z1, and C.vs.Z2). The analyses showed that most of the genes were closely related to the pathways of phenylpropanoid biosynthesis, phytohormone signalling, carbohydrate metabolism, transcription factors, and secondary metabolites.

### 4.1. Expression of Genes Related to Melatonin Synthesis in Germinating Barley under ZnSO_4_ Stress

In the biosynthesis of melatonin, tryptophan plays the role of the starting molecule. Firstly, TDC is responsible for catalysing the decarboxylation reaction of tryptophan to produce tryptamine. Next, T5H converts tryptophan to serotonin. Serotonin is then catalysed in the chloroplast by SNAT to produce N-acetylserotonin. Next, ASMT or COMT catalyses N-acetylserotonin into melatonin in the cytoplasm. In addition, serotonin can also produce melatonin through another pathway. That is, serotonin is methylated to 5-methoxytryptamine in the cytoplasm by ASMT (or by simultaneous interaction with COMT). Subsequently, 5-methoxytryptamine is acetylated by SNAT in the chloroplast, ultimately producing melatonin. Together, these two pathways constitute a complex biosynthetic process from tryptophan to melatonin [8].

**Figure 6:**
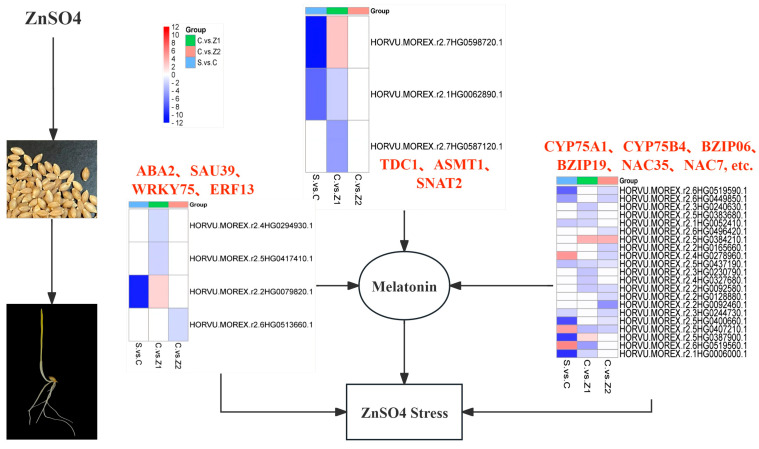
Schematic diagram of the changes in melatonin synthesis–related genes and the response mechanism of ZnSO_4_ stress resistance under different concentrations of zinc sulphate stress.

In our study (Figure 6 and Table 1), the expression pattern of the gene *TDC1* showed an inconsistent trend. In the S.vs.C comparison, the expression of *TDC1* appeared to be down-regulated; however, in the C.vs.Z1 comparison, its expression was up-regulated. We speculate that this change in expression pattern may be related to fluctuations in melatonin content. We analysed the gene *ASMT1*. The results showed that *ASMT1* was down-regulated in both the S.vs.C and the C.vs.Z1 comparisons. This finding suggests that melatonin synthesis is mainly dependent on *ASMT1* catalysing the conversion of serotonin to melatonin during germination or under a low concentration of ZnSO_4_ stress. This finding provides a new perspective from which to deeply understand the mechanism of melatonin synthesis and its important role in plant response to environmental stress.

In addition, in the C.vs.Z1 comparison, we observed down-regulation of the gene *SNAT2* alone, suggesting that our barley may provide more substrates for melatonin synthesis by regulating the expression of *SNAT2* in the face of zinc sulphate stress (Figure 6 and Table 1). However, it is noteworthy that we did not observe the expression of the abovementioned genes related to melatonin synthesis in the comparison of C.vs.Z2. This may be due to the fact that the melatonin synthesis pathway in barley becomes more complex when it is stressed by higher concentrations of ZnSO_4_ and may involve more currently unknown regulatory mechanisms and gene expression changes.

### 4.2. Oxidative Stress Involved in Germinating Barley under ZnSO_4_ Stress

Under normal physiological conditions, plants produce appropriate amounts of reactive oxygen species (ROS) and maintain their dynamic balance, which is essential for normal plant growth and development. However, when plants are exposed to abiotic stresses, especially salt stress, ROS production increases significantly, leading to oxidative stress and cellular damage, which in turn inhibits the seed germination process [38]. During the transition from the dry seed to the germination stage, there is a gradual increase in the production of ROS; in response to this, plants activate their antioxidant enzyme systems, such as peroxidase and ascorbate peroxidase, in order to protect against deleterious oxidation induced by metal stress [39]. As a free radical scavenger, melatonin effectively combats harmful reactive ROS and serves as a protector against abiotic stress in plants [40]. For instance, melatonin synthesis genes in cassava—*MeTDC1*, *MeTDC2*, etc.—have been identified as playing a role in ROS and defence signal transduction [41]. In the case of mung bean seedlings exposed to salt stress, the application of exogenous melatonin enhances the plant’s antioxidant defence system, increases photosynthetic activity, and mitigates oxidative damage caused by ROS [42].

The phenylpropanoid biosynthesis process has a critical ROS-scavenging function when plants are subjected to salt stress [43]. In our study, changes in the expression of related enzymes were observed at different stages of ZnSO_4_ stress. CAD and CCR genes are key players in the process of lignin synthesis in plants, which plays a significant role in the stabilisation of the plant cell wall, the enhancement of the plant’s resistance to diseases, and the improvement of abiotic stress tolerance [44,45]. The expression of two probable cinnamyl-alcohol dehydrogenase 5 (*CAD5*) was increased in Z1 compared to C, while two cinnamoyl-CoA reductase 1 (*CCR1*) were also elevated in Z2 (Table S6). These enzymes were induced by ZnSO_4_ stress, suggesting that they play an important role in plant response to this environmental stress. In addition, peroxidase (POD) is involved in the production and scavenging of hydrogen peroxide (H_2_O_2_) in plants. As a key antioxidant, it plays an important role in clearing excess reactive oxygen species (ROS) generated by abiotic stress [46]. Our study revealed that under ZnSO_4_ stress, several POD-related genes, including *PER15*, *PER2*, *PER51*, *PER70*, and *PER19*, were induced to express (Table S6). These genes may mitigate the oxidative stress induced by salt stress through the stimulation of melatonin, thereby enhancing the tolerance of barley to ZnSO_4_ stress [47].

The expression of key genes in flavonoid biosynthesis, such as *PAL* and *FLS*, was induced, which helped to enhance the salt tolerance of the plant and, consequently, the plant’s ability to respond to environmental stresses [48]. Injecting 0.1 mM melatonin into the peels of citrus fruits can enhance the expression of key genes involved in several flavonoid biosynthesis pathways, including 4-coumaroyl CoA ligase (*4CL*), flavone synthase (*FNS*), and flavonoid hydroxylases (*FHs*) [49]. Similarly, soaking grape berries in a 50 μM melatonin solution not only improves the plants’ disease resistance but also enhances flavonoid biosynthesis [50]. The study found that treating cabbage seeds with 10–1000 μM melatonin for 12 h could up-regulate the expression of crucial structural genes in the anthocyanin biosynthesis pathway in cabbage sprouts, including phenylalanine ammonia lyase (*PAL*), cinnamate 4-hydroxylase (*C4H*), chalcone synthase (*CHS*), chalcone isomerase (*CHI*), and flavanone 3-hydroxylase (*F3H*). This treatment reduced the levels of reactive ROS in plants and improved their tolerance to environmental stress [51]. We observed that several key genes, including *CHI*, were expressed under Z1 and Z2 conditions compared to the control (C). Additionally, the genes *ANR* (Anthocyanidin reductase ((2S)-flavan-3-ol-forming)), *CYP75A1* (Flavonoid 3′,5′-hydroxylase 1), *CYP75B4* (Flavonoid 3′-monooxygenase), and *AGCT2* (Agmatine coumaroyltransferase-2) were specifically induced under Z2 conditions. Notably, in the comparison of S.vs.C, the expression of most of these genes exhibited a down-regulation trend, suggesting that these genes may be activated during seed germination. Furthermore, the high expression of these genes under ZnSO4 stress may indicate their positive role in enhancing barley’s resistance to salt stress, which is associated with varying melatonin levels under ZnSO_4_ stress (Table S6). The *CYP75B1* gene was reported to significantly up-regulate the rutin synthesis process in Syringa oblata Lindl, suggesting that it may be a key regulatory gene in the flavonoid biosynthetic pathway [52]. In our study, the *CYP75A1* gene was induced in Z2 compared to C. Plants rely on their defence mechanisms in the face of abiotic stresses; these mechanisms usually involve metabolic adjustments that allow survival in unfavourable environments. One of these protective mechanisms is the production of melatonin, which acts as a free radical scavenger in plants, contributes to the neutralisation of ROS, and enhances other existing enzymatic and non-enzymatic antioxidant activities, thereby increasing abiotic stress tolerance [53]. Thus, these genes with antioxidant effects play an important role in the zinc-stress response, while their high expression is closely associated with melatonin production.

### 4.3. Phytohormone Signalling Involved in Germinating Barley under ZnSO_4_ Stress

Plant hormones, including growth hormone (IAA), abscisic acid (ABA), and ethylene, are indispensable substances in plant growth and development. These hormones are not only involved in the regulation of plant growth and developmental processes but are also capable of mediating salinity signalling, which in turn regulates plant adaptation to salt stress [54]. Abscisic acid (ABA), as a key phytohormone, plays an important role in plant growth and development. Under salt-stress conditions, ABA can regulate the expression of many salt-stress-responsive genes, such as MYB, ERY, and other transcription factors, thereby improving the tolerance of plants to salt stress [55]. It was found that the addition of sulphate affected ABA synthesis in Arabidopsis seedlings. In Arabidopsis thaliana, as the sulphate content increased, the ABA content increased accordingly, suggesting that sulphate may regulate the plant response to salt stress by affecting ABA synthesis [56]. In addition, the expression of melatonin-related genes in rice, such as *OsT5H*, *OsTDC1*, and *OsASMT1*, is usually regulated by multiple plant hormones, including ABA and jasmonic acid (JA). Moreover, these genes are regulated by various abiotic stresses and pathogen infections [57]. Melatonin has an important antioxidant role in plants, and it may co-regulate plant adaptation to salt stress through interactions with other phytohormones. Zeaxanthin epoxidase (*ABA2*) is involved in the process of ABA biosynthesis [58]. The *ABA2* gene was significantly induced in Z1 compared to C (Figure 6) and may enhance the adaptation of barley to ZnSO_4_ stress by increasing the content of ABA. However, melatonin synthesis is negatively regulated by ABA synthesis [59]; therefore, the up-regulation of the *ABA2* gene in Z1 may have led to a decrease in melatonin content.

Auxin (IAA) is an indole derivative that shares tryptophan as a synthetic precursor with melatonin. Research indicates that melatonin can enhance the IAA content in Arabidopsis seeds by up-regulating the expression of IAA-responsive genes, such as *IAA3* and *IAA13* [60]. Our findings reveal that, compared to C, the expression of Auxin-responsive proteins (*IAA13*) was induced under Z2 conditions, which may be associated with the elevated melatonin content observed in Z2 [61]. In addition, nine IAA family genes were down-regulated and two IAA genes were up-regulated in S.vs.C (Table S6). The *SAUR39* gene plays a negative regulatory role in IAA synthesis and transport. Endogenous melatonin is able to mediate growth hormone signalling and plays a key role in salt-stress response [62]. In our study, *SAUR39* was found to be expressed in Z1 (Figure 6) and may have contributed to the reduction in melatonin content in barley under ZnSO_4_ stress. The study demonstrated that soybean seedlings, upon reaching the V1 stage and being treated with 20 µM melatonin, exhibited an increased expression level of indole-3-acetic acid-amido synthetase (*GH3*), a gene integral to IAA synthesis. This finding indicates a strong relationship between melatonin and IAA synthesis [63]. Under C treatment conditions, several members of the *GH3* gene family, including *GH34*, *GH38*, *GH32*, and *GH31*, showed induced expression when compared to S. Furthermore, under Z2 treatment conditions, the *GH31* gene was found to be highly expressed in comparison to C. However, no significant expression changes in the *GH3* gene were observed in the comparison between C and Z1.

Melatonin not only regulates seed germination and root and shoot growth but also participates in the plant senescence process [64]. For example, the melatonin receptor (*CAND2*/*PMTR1*) found in Arabidopsis thaliana has been shown to regulate plant stomatal closure [65], which provides further evidence of the important role of melatonin in plant physiology. In addition, melatonin regulates plant hormone levels and the expression of stress-responsive genes under unfavourable conditions [66,67], which shows that it plays a central role in the plant stress response. Therefore, we hypothesised that the expression of phytohormone-regulated genes might be regulated by melatonin production. However, the pathways involved were not the same in barley under different concentrations of zinc sulphate stress, suggesting that melatonin production in barley under salt stress may be affected by a complex regulatory mechanism. This complex regulatory network may involve the interaction of multiple phytohormones and signalling pathways to achieve the fine regulation of plant growth and development and the stress response.

### 4.4. Transcription Factors Involved in Germinating Barley under ZnSO_4_ Stress

Transcription factors such as bHLH, ERF, NAC, WRKY, MYB-related, and MYB have been reported to be involved in the seed germination process and have been regarded as key factors in plant resistance to salt stress [32]. Meanwhile, melatonin has also been found to induce the expression of genes such as WRKY, MYB, and NAC [68], further highlighting the importance of these transcription factors in the plant stress response. In our study, a total of 1768 differentially expressed genes (DEGs) from 55 transcription factor (TF) families were identified via a comparison of the S.vs.C groups. Notably, these DEGs were mainly enriched in the bHLH, ERF, NAC, C2H2, WRKY, B3, MYB-related, and MYB families. We identified 24 major transcription factors that were salinity-responsive in the C.vs.Z1 comparison, suggesting that they may be directly involved in the response to and regulation of salt stress. Moreover, we identified 22 major transcription factors as possible regulators in response to zinc sulphate toxicity in the C.vs.Z2 comparison (as shown in Table S6).

In cassava, the *WRKY 75* transcription factor has been shown to interact with the melatonin synthases *TDC2* and *ASMT2/3*, and this interaction enhances the endogenous melatonin content in cassava [69]. In our study, the expression of *WRKY 75* increased under C treatment conditions and down-regulated in Z1 (Figure 6); this is a difference that may contribute to the lower melatonin content in Z1 compared to C. Studies have revealed that the *OsWRKY45-2* allele negatively regulates the response to salt stress in rice [70]. Our observations indicated that the expression of *WRKY45-2* was inhibited under Z1 and Z2 treatments, while it was induced under C. This finding suggests that the inhibition of *WRKY45-2* may enhance barley’s tolerance to ZnSO_4_ stress. Additionally, the expression level of *OsWRKY45* was significantly induced by melatonin [71]. Therefore, *WRKY45-2* is induced in condition C and may contribute to melatonin synthesis. Basic leucine zipper (bZIP) transcription factors play an important role in plants; they are involved in the regulation of processes such as seed maturation, development, and signalling and can increase plant tolerance to abiotic stresses [72]. In *Arabidopsis thaliana*, the *bZIP19* and *bZIP23* genes are able to regulate the transcriptional levels of zinc homeostasis in response to changes in zinc status in cells [73]. Under drought stress, soybean seedlings that were treated with 1 mM melatonin exhibited increased expression levels of the transcription factors *GmbZIP* and *GmDREB2* [74]. In our study, two genes, *bZIP06* and *bZIP19*, showed a trend of down-regulation only in the comparison of C.vs.Z1 (Figure 6), whereas no changes in the bZIP family transcription factors were detected in the comparisons of C.vs.Z2 or S.vs.C. It has been reported that in *Arabidopsis*, *bZIP19* primarily promotes the expression of genes, such as *ZIP9*, under conditions of zinc deficiency [75]. Consequently, it remains unclear whether the transcription factor bZIP is involved in the synthesis of melatonin and whether it responds to ZnSO_4_ stress.

The NAC gene, which is one of the plant-specific transcription factors, is widely recognised as a gene that enhances plant tolerance to abiotic stresses [76]. In our study, we observed that *NAC35* was significantly down-regulated in both the S.vs.C and the C.vs.Z1 comparisons; however, no change in its expression was found in the C.vs.Z2 comparisons. In contrast, the *NAC7* gene showed a greater tendency to be induced in Z2 compared with C (Figure 6), but no change in its expression was detected in Z1. These results imply that the expression of *NAC7* may be specifically induced under high salt stress, whereas *NAC35* may play a different role during seed germination or in the early salt-stress response. In addition, we found that other transcription factors, including *BHLH35*, *BHLH70*, *MYBAS2*, and *MYBP*, were also significantly down-regulated in C.vs.Z1. The down-regulation of these transcription factors may have affected the response and adaptation of the plants to salt stress. However, it remains unclear whether there is a connection to melatonin, and the specific mechanism of action needs to be revealed by further studies.

The growth hormone–induced overexpression of the Ethylene-responsive transcription factor *ERF13* was reported to inhibit the emergence of lateral roots in plants [77], while in another study, *MeRAV1* and *MeRAV2* of the Apetala 2/ethylene response factor (*AP2*/*ERF*) gene family were found to positively regulate melatonin biosynthesis genes and endogenous melatonin levels. Further studies revealed that *MeRAV1* and *MeRAV2* are direct transcriptional activators of three melatonin biosynthesis genes in cassava [78]. In our study, the expression of *EFR13* was induced in Z2 rather than C (Figure 6), suggesting that *EFR13* may act as a key regulator involved in the response of barley to Zn toxicity in synergy with hormones such as ethylene. Meanwhile, the induced expression of *EFR13* may also have a positive effect on melatonin synthesis, considering the promotional effect of *MeRAV1* and *MeRAV2* on melatonin synthesis. This finding provides a new perspective from which to further understand the roles of phytohormones and melatonin in plant stress response.

## 5. Conclusions

This study employed transcriptomic analysis to elucidate the response and tolerance mechanisms of melatonin in germinating hull-less barley seeds subjected to zinc sulphate stress. Although zinc sulphate stress inhibited barley growth, it concurrently elevated the levels of endogenous melatonin. The DEGs associated with stress were primarily involved in biological pathways related to oxidative stress, plant hormone signalling, and secondary metabolite biosynthesis. Notably, alterations in genes such as *TDC1*, *ASMT1*, *SNAT2*, *WRKY 75*, and *EFR13* indicated that melatonin plays a crucial role in detoxifying zinc sulphate and enhancing stress tolerance.

## Figures and Tables

**Table 1:** Expression of genes involved in melatonin synthesis under ZnSO_4_ stress.

Gene ID	Gene Name	Log_2_FoldChange		
		S.vs.C	C.vs.Z1	C.vs.Z2
HORVU.MOREX.r2.7HG0598720.1	*TDC1*	−11.38	3.00	0.00
HORVU.MOREX.r2.1HG0062890.1	*ASMT1*	−6.92	−2.30	0.00
HORVU.MOREX.r2.7HG0587120.1	*SNAT2*	0.00	−4.66	0.00

## Data Availability

All data generated in this study are included in the article and are available from the corresponding author upon reasonable request.

## Supplementary Materials

The following supporting information can be downloaded at: https://www.mdpi.com/article/10.3390/genes15081077/s1, Figure S1: Venn diagram of Upset; Table S1: Read count and FPKM for all samples; Table S2: Cluster table of differentially expressed genes; Table S3: GO functional enrichment analysis of differentially expressed genes in the S.vs.C comparison group; Table S4: GO functional enrichment analysis of differentially expressed genes in the C.vs.Z1 comparison group; Table S5: GO functional enrichment analysis of differentially expressed genes in the C.vs.Z2 comparison group; Table S6: Expression of melatonin synthesis-related genes under different concentrations of zinc sulphate stress.
