# Supplementary figures and images for: Revealing the Effects of Zinc Sulphate Treatment on Melatonin Synthesis and Regulatory Gene Expression in Germinating Hull-Less Barley through Transcriptomic Analysis
Source: Genes (Basel). 2024 Aug 15;15(8):1077. doi: 10.3390/genes15081077 (PMC11354046; doi:10.3390/genes15081077)

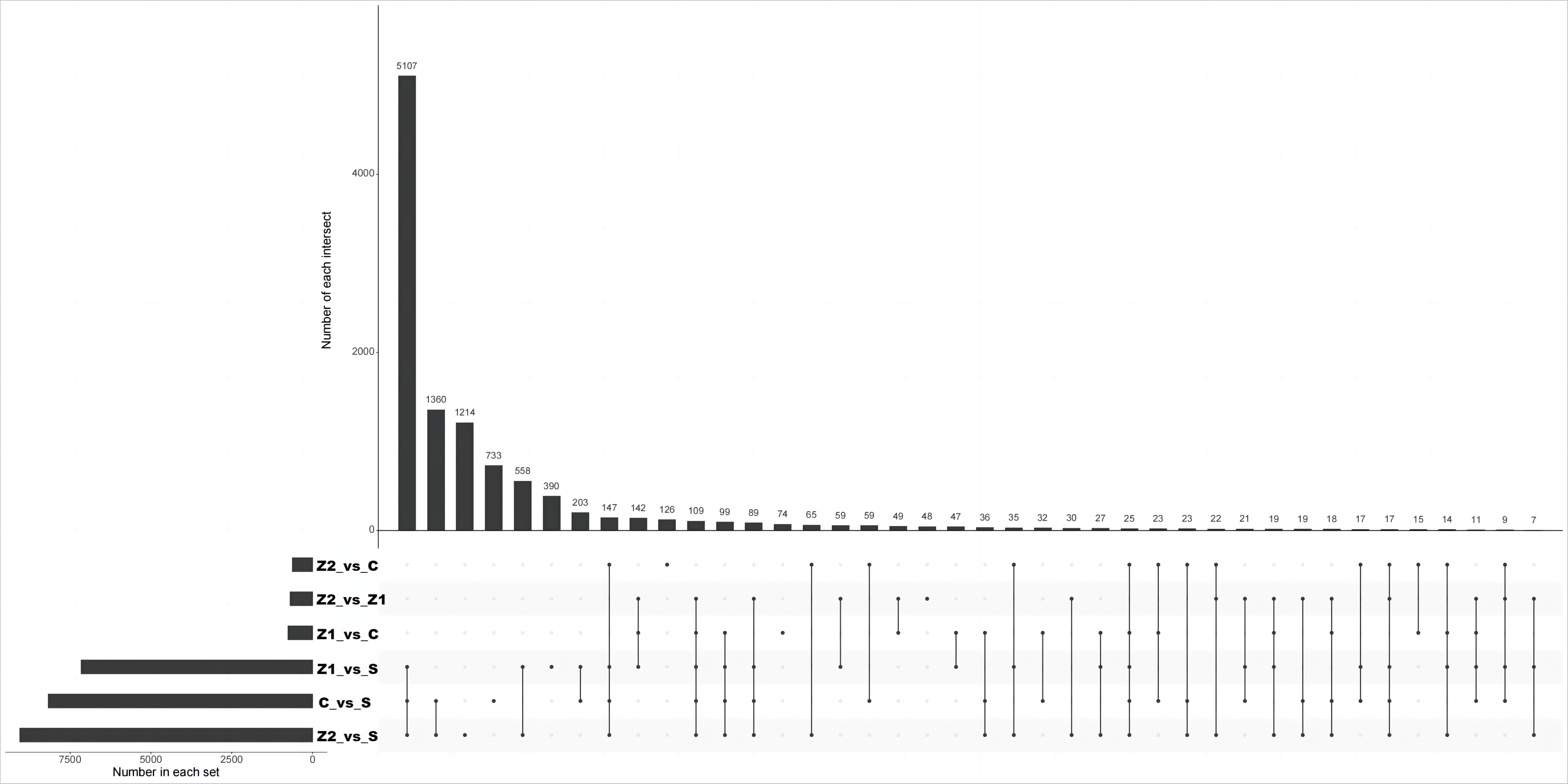

Supplement: Supplementary file 1 [file genes-15-01077-s001.zip › Supplementary figures/Figure S1 venn.png]
